# Development of a Method to Implement Whole-Genome Bisulfite Sequencing of cfDNA from Cancer Patients and a Mouse Tumor Model

**DOI:** 10.3389/fgene.2018.00006

**Published:** 2018-01-23

**Authors:** Elaine C. Maggi, Silvia Gravina, Haiying Cheng, Bilal Piperdi, Ziqiang Yuan, Xiao Dong, Steven K. Libutti, Jan Vijg, Cristina Montagna

**Affiliations:** ^1^Department of Genetics, Albert Einstein College of Medicine, New York, NY, United States; ^2^Department of Oncology, Montefiore Medical Center, Albert Einstein College of Medicine, Bronx, NY, United States; ^3^Department of Surgery, Albert Einstein College of Medicine, New York, NY, United States; ^4^Department of Ophthalmology and Visual Science, Albert Einstein College of Medicine, New York, NY, United States; ^5^Obstetrics & Gynecology and Women’s Health, Albert Einstein College of Medicine, New York, NY, United States; ^6^Department of Pathology, Albert Einstein College of Medicine, New York, NY, United States

**Keywords:** cell-free DNA, cfDNA, DNA methylation, non-invasive blood based screening, biomarker, pancreatic cancer, circulating DNA, mouse cfDNA

## Abstract

The goal of this study was to develop a method for whole genome cell-free DNA (cfDNA) methylation analysis in humans and mice with the ultimate goal to facilitate the identification of tumor derived DNA methylation changes in the blood. Plasma or serum from patients with pancreatic neuroendocrine tumors or lung cancer, and plasma from a murine model of pancreatic adenocarcinoma was used to develop a protocol for cfDNA isolation, library preparation and whole-genome bisulfite sequencing of ultra low quantities of cfDNA, including tumor-specific DNA. The protocol developed produced high quality libraries consistently generating a conversion rate >98% that will be applicable for the analysis of human and mouse plasma or serum to detect tumor-derived changes in DNA methylation.

## Introduction

Non-invasive blood based screening is emerging as a promising alterative to traditional tissue biopsies in the management of cancer patients. They have garnered much research focus and recently made their entrance in clinical settings as diagnostic tools with great promise for early disease diagnosis or prognosis (Maggi et al., 2016). Fluid phase biopsies profit from the release of tumor cellular components into the bloodstream or other biological fluids. Molecular profiling of DNA offers the potential to discover unique, novel biomarkers for cancer screening with the ultimate goal to diagnose cancer and/or its recurrence as an alternative to conventional methods (e.g., imaging). In addition, non-invasive blood based screenings can be performed when traditional tissue biopsies are not feasible or when the collected tissue is not sufficient for diagnostic analysis and testing of biomarkers of interest. This is a common scenario in lung cancer diagnosis and clinical follow up (Ilie et al., 2014).

One type of material that can be isolated from the blood and analyzed at the molecular level is cell-free DNA (cfDNA). cfDNA is composed mainly of short DNA fragments of ∼160 bp and its multiples, likely generated by extrusion of DNA from apoptotic cells (Mouliere and Rosenfeld, 2015). Small fractions of longer fragments can also be present, and likely derive from necrosis at the tumor site (Stroun et al., 1989). cfDNA can be isolated from plasma or serum and other biological fluids and used for high throughput genomic analysis (Jahr et al., 2001; Cooke and Campbell, 2012). cfDNA isolation from serum usually produces higher yields than plasma (Lee et al., 2001), however, serum derived cfDNA is particularly susceptible to genomic DNA contamination from non-tumor cells likely due to leukocyte lysis (Warton et al., 2014) making it difficult to detect disease specific genomic or epigenomic changes. In disease-free individuals cfDNA can be isolated, but its concentration is generally low (Spindler et al., 2014). Certain disease states such as cancer are associated with high cfDNA concentrations, making its analysis more sensitive (Leon et al., 1977; Giacona et al., 1998). The use of cfDNA for biomarker discovery has concentrated on two main areas of investigation: mutational analysis and DNA methylation analysis. Mutational analysis has been, thus far, more prominent and diagnostic tests developed utilizing this new technology are already proposed in clinical settings (Maggi et al., 2016; Kwapisz, 2017). Currently the use of cfDNA for prenatal testing has been accepted, but cfDNA based tests for the identification of cancer pathogenic actionable driver mutations in patients with cancer and those at high risk of developing tumors are rapidly emerging (Qin et al., 2016; Taylor-Phillips et al., 2016).

Cancer precision medicine can now be carried out by targeting all genes in whole exome sequencing or a panel of genes known to be often mutated in a given tumor. However, cfDNA is less suitable for such an approach since tumor-derived cfDNA is mixed with excess of non-tumor cfDNA making the identification of specific mutations particularly challenging. While cancer patients generally have higher cfDNA levels (Stroun et al., 2001), the ratio between tumor derived cfDNA and that from non-tumor cells varies widely, from 0.01% to more than 90% (Schwarzenbach et al., 2008, 2009, 2011; Salvianti et al., 2012; Qin et al., 2016). This variability is likely a reflection of tumor burden, stage, response to therapy and other physiological tumor-related processes. The power of cfDNA as a non-invasive biomarker, however, lies in early detection, monitoring response to therapy, or assessing recurrence, all clinical conditions associated with a lower frequency of tumor-derived cfDNA and precisely when its detection is the most challenging. Technical limitations that can further decreased the ratio of tumor derived and non-tumor cfDNA however also exist. For example high molecular weight DNA can be present after cfDNA extraction originating from contaminating blood derived non-tumor cells retained in the plasma or serum after separation prior tissue banking. Serum derived cfDNA has been reported more susceptible to this shortcoming than plasma derived cfDNA (Lee et al., 2001).

An attractive alternative to overcome the limitations of mutational profiling is to analyze cfDNA for methylation changes instead. Functionally relevant DNA methylation changes found in tumor cells, such as those observed at CpG islands and shores, span many CG sites and are commonly referred to as differentially methylated regions (DMRs) (Irizarry et al., 2009). The presence of multiple epigenetic changes within small genomic regions facilitates their mapping over single nucleotide changes responsible for tumor suppressor inactivation or oncogene gain of function. Hence, such genomic regions are much more likely to be detected in cfDNA than a single base pair change. In addition, DNA methylation changes observed in the tumors have been detected also in neighboring non-mutated, histologically normal cells as well as in the stroma (Umbricht et al., 2001). The presence of DMRs in seemingly normal cells are likely to be reflected in cfDNA, therefore potentially increasing their frequency over non-tumor-derived cfDNA. Additionally, intra-tumor heterogeneity has been widely investigated and it is now recognized as critical for tumor evolution and a major modulator of clinical outcome (Turner and Reis-Filho, 2012; Hu et al., 2017). Because most tumor cells bear only one or few driver mutations (Vogelstein et al., 2013), the respective frequency of each single nucleotide change in tumor-derived cfDNA is further diluted. By contrast, DNA methylation changes have been shown to converge to more constant genomic regions, even when the driver mutations are different (Brocks et al., 2014). Therefore, different clones within the same tumors as well as their respective metastatic sites, are more likely to show similar DNA methylation changes regardless of which specific driver mutation originated the tumor (Esteller, 2002). As a result, if sequenced at high coverage, altered methylation detected by bisulfite sequencing is likely to be a more robust and more sensitive biomarker than mutations.

While DNA methylation profiling of cfDNA is an attractive approach for clinical diagnostic, major technical challenges associated with this analysis have thus far limited its expansion. The aforementioned contamination of high molecular weight DNA originating from lysed lymphocytes and other circulating non-tumor cells limits the analytical sensitivity of molecular based approaches. While stringent standard operating procedures for bio fluid collection, processing and storage can mitigate this problem, this remains a significant obstacle especially for serum-derived cfDNA and for bio-banked material that may not promptly undergo serum or plasma isolation (Bronkhorst et al., 2015). Moreover, treatment of cfDNA with sodium bisulfite results in further fragmentation and significant loss of starting material during desulfonation and purification procedures. As a result, most of the cfDNA studies reported thus far employ PCR based approaches rather than whole genome sequencing analysis (Cheuk et al., 2017).

Here, we present a practical procedure for whole-genome bisulfite sequencing (WGBS) of ultra-low amounts of cfDNA from the serum or plasma of patients diagnosed with pancreatic neuroendocrine tumors (PNETs) or lung cancer as well as from a mouse models of pancreatic ductal adenocarcinomas (PDACs) and healthy human and mouse controls.

## Materials and Methods

### Plasma or Serum Collection and cfDNA Isolation

Human samples (lung cancer patients *n* = 9, pancreatic neuroendocrine patients *n* = 7, and controls *n* = 6) were obtained from plasma/serum biobanks established at Albert Einstein College of Medicine. All lung cancer blood samples were obtained from patients with stage IV adenocarcinomas, some with known mutations in KRAS (samples #3 and #7) or EGFR (samples #4, #6, and #8) (**Table 1**). PNET blood samples were obtained from patients with confirmed PNETS at varying stages of disease progression and treatment status (**Table 2**). Control samples were taken from individuals with no known cancer or other diseases. This study was approved by the Albert Einstein College of Medicine Committee on Clinical Investigations (CCI#2007-433 and 09-06-173). Blood from control and lung cancer patients (∼10 ml), was collected in EDTA tubes and kept at room temperature for less than 6 h before centrifugation at 2000 × *g* for 30 min at 4°C to separate the plasma fraction from the buffy coat and the erythrocytes. The plasma was aliquoted in ∼2 ml fractions to avoid multiple freeze-thaw cycles and stored at -80°C until use. Blood from PNET patients was collected in red top tubes and allowed to clot at room temperature for 30 min before centrifugation at 2000 × *g* for 30 min at 4°C. Serum was then collected and frozen at -80°C in ∼1 ml aliquots.

**Table 1:** Clinical information for lung adenocarcinoma patients.

Sample name	Gender	Age	Stage	Race	Treatment at collection	EGFR (y/n/nt)	Specific mutation
L1	M	68	IV	Multiracial	pem/bev maintenance	nt	n/a
L2	M	57	IV	Asian	Docetaxel	n	n/a
L3	M	74	IV	Asian	pem/bev maintenance	n	n/a
L4	F	66	IV	Multiracial	Erlotinib	y	Exon 18 (Q701L and G719A)
L5							
L6	F	76	IV	Multiracial	None (off erlotinib)	y	Exon 21 (L858R mutation)
L7	F	61	IV	White	Docetaxel	n	n/a
L8	F	62	IV	White	Erlotinib	y	Exon 19 (E746_A750del5 mutation)
L9	F	49	IIIA	White	Pre-treatment	y	Exon 19 del

**Table 2:** Clinical information for PNET patients.

Sample name	Gender	Age	Pathology report
P1	F	76	G1 – well-differentiated
P2	F	64	Low grade and high-grade component. 17 cm × 15 cm × 7 cm. Lymph nodes (-) (0/7)
P3	F	43	
P4	M	14	Well-differentiated, low grade
P5	F	54	Well-differentiated, 1.9 cm greatest dimension, grade 2%, ki-67 5%
P6	F	66	
P7	F	50	G1, 3.1 cm × 2.8 cm × 2.5 cm surgical resection margin is negative for tumor. Nine lymph nodes negative for tumor (0/9)

Mouse blood (∼0.2 ml per mouse per blood draw) was obtained from a pancreatic cancer mouse model and age matched controls (KPC; *LSL*-*p53*^R*172*H/+^; *LSL*-*Kras*^G*12*D/+^; *Pdx-Cre*). Blood was obtained from 12 individual mice older than 18 weeks of age (*n* = 6 KPC and *n* = 6 age matched controls) in two non-terminal blood draws from the submandibular facial vein with recovery time in between, collected in EDTA tubes and stored for a maximum of 24 h at 4°C before being processed as described above. Before cfDNA extraction, the two non-terminal blood draws from each mouse were combined (∼0.4 ml of blood per mouse), and two mice were pooled together (∼0.8 ml of total blood per sample set) producing ∼0.4 ml of plasma given that the plasma is about half of the blood volume. As result we extracted cfDNA from *n* = 3 KPC and *n* = 3 age matched controls.

### Isolation of cfDNA from Human and Mouse Plasma/Serum

To isolate the cfDNA we utilized the Qiagen QIAamp Circulating Nucleic Acid kit (Cat no./ID: 55114) with the modifications described in Section “Results.” For cfDNA purification we compared Solid Phase Reversible Immobilisation beads (SPRI) from Beckman Coulter (Agencourt AMPure cat#A63880) with the DNA Clean & Concentrator kit, Zymo Research (D4003T). Details of cfDNA purification are described in Section “Results.” All samples were analyzed using the bioanalyzer (Agilent, Santa Clara, CA, United States) to assess the distribution of cfDNA size using a high sensitivity DNA chip.

### Bisulfite Conversion, Library Construction, and Sequencing

Bisulfite converted libraries were generated using the Zymo Research Pico Methyl-Seq^TM^ Library Prep Kit (D5455). The libraries were multiplexed at five or six samples per lane and were run using 1 × 100 bp cycles on the Illumina MiSeq sequencer. Libraries concentrations were measured prior to sequencing using the KAPA Biosystems Library Quantification Kit for Illumina Libraries (Cat#07960140001).

### Data Analysis

After sequencing, the resulting fastq files were evaluated using FastQC0.11.2/java.1.7.0_67 (Andrews, 2010). Sequencing reads were aligned to the hg38 human genome or the mm10 mouse genome using the Bismark software package v0.14.5 (Krueger and Andrews, 2011). Heatmaps were generated using the heatmap function in the RStudio Package for the R bioconductor (RStudio-Team, 2015). Statistical analyses for group comparisons were performed using Prism (GraphPad Software, Inc., United States). Sequencing reads were visualized using the Integrative Genomics Viewer (IGV) (Robinson et al., 2011). Raw sequencing data can be downloaded trough the Sequence Read Archive (SRA) portal^1^ using the following bio projects IDs: human dataset PRJNA418597, mouse dataset PRJNA418769.

A detailed step-by-step protocol can be found in the Supplementary Materials.

## Results

### Purification of cfDNA from Contaminating High Molecular Weight DNA

Human samples from two different cancer types, PNETs and lung cancer, as well as healthy controls were used in this study (see **Tables 1**, **2** for details on the patient cohorts).

As a murine counterpart for human PNETs, we selected mice with the following genotype: KPC; *LSL*-*p53*^R*172*H/+^; *LSL*-*Kras*^G*12*D/+^; *Pdx-Cre*. KRAS and *p53* are mutated in ∼90 and ∼75% of human pancreatic cancer cases, respectively. *Kras* and *p53* double mutations in mice can reproduce the entire progression of PDAC, this model is known as the KPC model of pancreatic cancer (Weissmueller et al., 2014; Lennerz and Stenzinger, 2015). By ∼10 weeks all mice develop pancreatic intraepithelial neoplasia (PanIN) lesions with PDAC formation at ∼18 weeks of age; median survival is ∼5 months with most mice presenting advanced metastases (Hingorani et al., 2005). For this study blood was collected using a non-invasive approach, a submandibular facial vein draw. This particular sampling method allows for longitudinal studies of disease progression in individual animals. Because the maximum amount of blood that can be collected from each mouse is ∼0.2 ml (which yields only ∼0.1 ml of plasma for each draw time point), two independent non-terminal blood draws and two age and genotype matched samples were pooled to obtain 0.8 ml of blood generating ∼0.4 ml total plasma and used to isolate cfDNA to give three control samples and three KPC samples.

**FIGURE 1:**
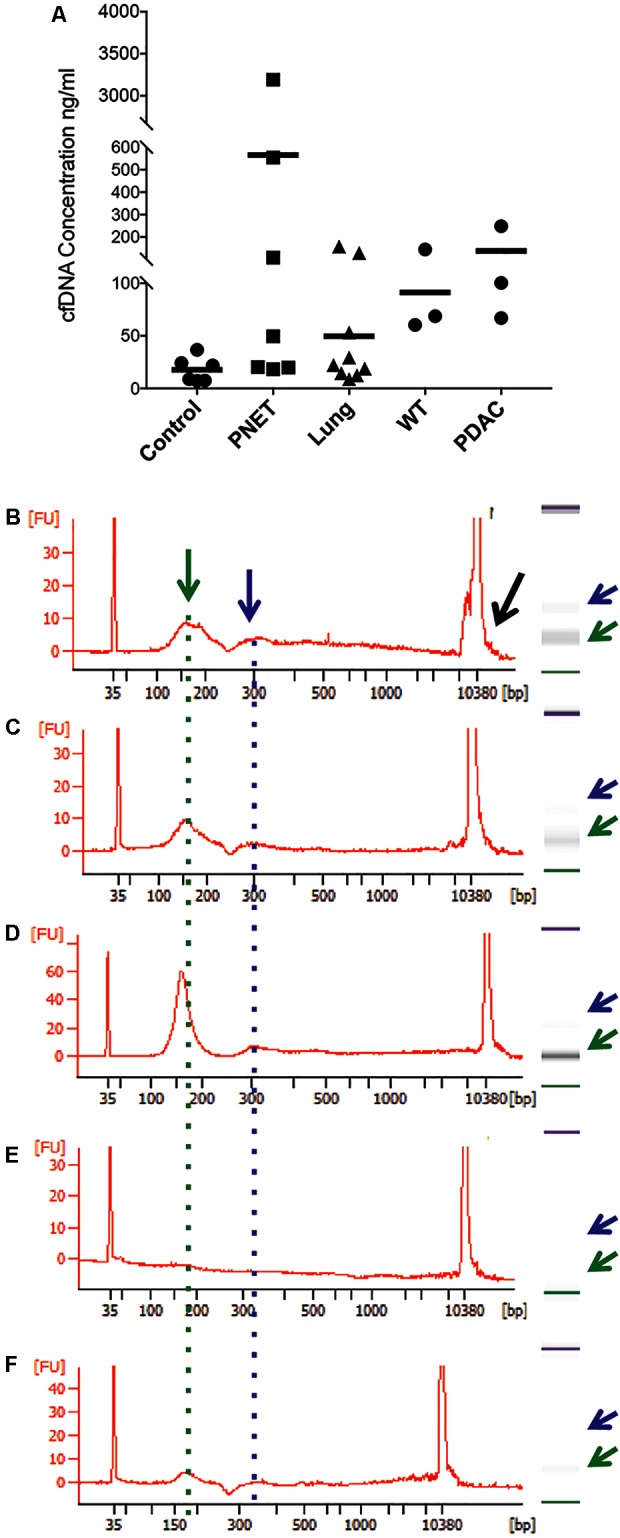
Representative bioanalyzer profiles of cfDNA. **(A)** The concentration of cfDNA per ml of plasma (human control and lung cancer and mice) or serum (human PNET) is shown for each of the samples analyzed. The horizontal bar indicates the average values for each sample set. **(B)** Healthy human control #6, **(C)** PNET patient #4, **(D)** lung cancer patient #9, **(E)** WT mouse #1, and **(F)** PDAC mouse #3. A peak with the highest DNA amount was detected around 160 bp (green arrow and dotted line). Secondary peaks at ∼320 bp (blue arrow and dotted line) were visible in all samples except the WT mouse control. Contaminating high molecular weight DNA was present in some samples (black arrow in **B**).

**FIGURE 2:**
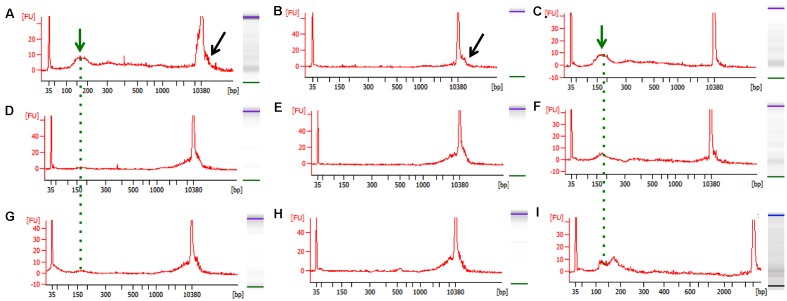
Representative bioanalyzer images of freshly isolated and bead-purified cfDNA. **(A–C)** Healthy human control, **(D–F)** WT mouse, and **(G–I)** PDAC mouse. The left columns depict the freshly isolated starting cfDNA (green arrow and dotted line) and the contaminating high molecular weight DNA (black arrow) evident by a wide peak. The middle column depicts the high molecular weight DNA removed from the same samples shown in **(A–G)** by the SPRI AMPure bead purification step after the 0.5X dilution step. The right column shows the purified cfDNA as recovered form the SPRI AMPure bead purification step after the 1.6X dilution step from the same samples shown in **(A–G)**. The desired cfDNA peak is visible ∼150–200 bp (green arrow and dotted line).

To isolate cfDNA we employed the QIAamp Circulating Nucleic Acid kit with the following modifications: (i) an additional wash with Wash buffer 1 (step 10) was added to ensure complete removal of the excess binding buffer and possible lysate contaminates, (ii) the elution buffer was heated temperature 40°C to maximize the recovery of DNA, (iii) and two elution steps (50 then 30 μl) were used. A fluorometric quantification (Qubit) was used to determine the DNA concentrations and used to estimate the concentration of cfDNA per ml plasma/serum (**Table 3**). The human control samples had relatively little variability, but the human cancer patients and mouse samples had highly variable cfDNA yield. As expected (Maggi et al., 2016), those with cancer had a generally higher concentration in both mice and humans (particularly in PNETs), though the differences were not statistically significant, likely due to low sample size (**Figure 1A**). Since the cfDNA of the lung cancer cohort was plasma derived and that of the PNETs was serum derived we compared the cfDNA concentration between the two cancer groups given the previously reported higher cfDNA yield obtained when processing serum (Lee et al., 2001). Indeed, the mean cfDNA yield of the serum derived PNETs samples was 656.9 ng/ml of blood, while the plasma derived yield was 49.5 ng/ml of blood. These differences were statistical significant (*t*-test, *p* < 0.00001). Even when the PNET sample with the highest cfDNA concentration per ml of blood was removed (PNET sample #1, 3191.1 ng/ml) the difference between the cancer groups remained statistically significant (*t*-test, *p* = 0.0015). These results are in concordance with previous reports indicating higher cfDNA yield from serum samples; however, intrinsic differences between pancreatic and lung cancer biology affecting cfDNA release into the bloodstream cannot be excluded since a direct comparison between serum and plasma derived cfDNA from the same cohort was not possible. Interestingly, as previously reported (Cheng et al., 2009; Cortese et al., 2015; Hamaguchi et al., 2015) the WT mice had significantly higher cfDNA levels per ml of plasma than control human samples (*p* = 0.0061). The bioanalyzer profiles of fragment size distribution revealed a peak of ∼150–200 bp in the human samples with some having a small additional peak around 350 bp (**Figures 1B–D**). The peak of ∼150–200 bp in the mouse samples was less pronounced than in the human samples, reflecting the low amount of blood that can be drawn from the mouse while maintaining its viability for further longitudinal studies (**Figures 1E,F**). Some of the samples, those with the highest concentrations, had contamination of higher molecular weight bands around the 10,380 bp marker (**Figure 1B**, black arrow). Components of cfDNA include DNA shed by normal cells undergoing apoptosis in healthy individuals, but both necrosis and apoptosis of tumor cells and circulating tumor cells, and active secretion of DNA by living cells contribute to cfDNA in cancer patients (Snyder et al., 2016). While in theory, tumor cells can produce high molecular weight DNA and fragmented DNA, ∼90% of cfDNA is commonly fragmented to a mean length of ∼180 bp as demonstrated by the presence of tumor specific chromosomal, genetic or epigenetic alterations in the smaller fraction (Shu et al., 2017). Therefore, the presence of traces of high molecular weight DNA are very likely derived by contaminating non-tumor cells and can potentially reduce the sensitivity of DNA methylation profiling due to an undesirable increase in the amount of DNA from non-tumor origin in the sample. In our sample set the bioanalyzer profiles revealed a wide peak ∼10,380 bp overlapping with the high molecular weight marker (**Figure 2A**, black arrow). A wider peak suggestive of high molecular weight contamination was also seen in the mouse control cfDNA samples (**Figure 2D**) and the PDAC cfDNA samples (**Figure 2G**). We therefore proceeded to test two different methods for removing the contaminating traces of high molecular weight genomic DNA. We tested a PCR purification cleanup column method (DNA Clean & Concentrator kit) in parallel with the use of SPRI AMPure beads at differing concentrations to achieve enrichment of the insert size of interest (∼150–200 bp). Two cfDNA human samples were pooled to obtain sufficient material for multiple comparisons and then equally divided to ensure equivalent amounts of starting DNA. The manufactures protocol was followed for the column purification with a 1:6 dilution of sample to binding buffer being used with the DNA Clean & Concentrator kit to allow only binding of smaller molecular weight DNA fragments. Two AMPure bead steps were used in the size selection protocol with the first being a 0.5X beads to sample volumetric ratio step to remove the large genomic DNA followed by 1.6X beads to sample volumetric dilution ratio with the goal to bind the desired cfDNA fragments. At the completion of the protocol the Qubit was used to measure the recovered DNA. We recovered about 30% more cfDNA from the SPRI AMPure beads (0.92 ng/μl from column vs. 1.30 ng/μl from beads) when compared to the DNA Clean & Concentrator kit, which is a column based purification approach and, as apparent from these results, causes cfDNA loss.

**Table 3:** cfDNA concentrations in plasma or serum (ng/ml).

Sample	Control (plasma)	PNET (serum)	Lung cancer (plasma)	WT mice (plasma)	PDAC mice (plasma)
1	8.8	3191.1	22.3	145.0	100.3
2	7.5	554.1	18.8	68.6	66.9
3	7.3	49.6	53.0	60.3	248.0
4	21.8	18.2	128.8		
5	36.7	108.2	8.8		
6	24.1	19.9	12.5		
7		20.2	29.3		
8			14.4		
9			157.6		
Average	17.7	565.9	49.5	91.3	138.4
*SD*	10.9	1173.6	55.2	46.7	96.4

Since the SPRI AMPure bead purification resulted in higher DNA recovery yield we proceeded to test the efficiency of high molecular weight DNA removal using the bead purification protocol on one additional human cfDNA sample that we spiked with 10% genomic DNA. To ensure that the protocol would work equally well using murine samples, which contained far less starting material and a small cfDNA peak at ∼150–200 bp, we also carried on the same SPRI AMPure beads based purification protocol using the murine cfDNA samples. In this case we omitted the genomic DNA spike since we specifically selected murine samples that showed visible contaminating high molecular weight DNA in the bioanalyzer profile (**Figures 2D,G**). The results from the AMPure bead protocols with two size selections (beads concentration 0.5X followed 1.6X) are summarized in **Table 4**. This purification method allows for a sensitive estimation of large versus small cfDNA fractions. For example, from the human sample that was isolated from a control individual (C1) we recovered 100% of starting material. Since we spiked in 10% high molecular weight DNA our eluted C1 DNA contained 90% of patient derived cfDNA. Of this, after the SPRI AMPure beads size selection, 75% of the DNA was eluted in the smaller DNA fraction and 25% was eluted with the high molecular DNA fraction. This high molecular DNA fraction included 10% spike in DNA and 15% C1 derived high molecular DNA. Therefore based on these calculations, we estimated that about 75% of the eluted material was cfDNA and 25% was large genomic contaminating DNA (15% endogenous and 10% added spike). The recovery rate for the mouse samples was lower with about 81% of the WT DNA recovered of which ∼45% was cfDNA and ∼55% was genomic DNA. In the PDAC murine model we recovered ∼66% of the total input DNA of which ∼56% was cfDNA and 44% was genomic DNA. The samples were run on the bioanalzyer to confirm the efficiency of the size selection purification. As shown by the representative images in **Figure 2** the high molecular weight contaminating DNA remained in the 0.5X SPRI AMPure beads fraction as shown by the wide peak (**Figures 2B,E,H**). The purified cfDNA eluted from the beads with the 1.6X fraction was clearly visible in the bioanalyzer profile at ∼150–200 bp size (**Figures 2C,F,I**, dotted green line), while the marker peak at 10,380 bp is now narrow as a consequence of the removal of the contaminating high molecular weight genomic DNA (of note in **Figure 2** panels B,C are the elution of the sample shown in panel A; panels E,F depict the elution of sample D; and panels H,I depict the elution of sample G). This purification step also allows for cfDNA concentration in a smaller volume, enabling the maximum amount of starting material to be used for library generation.

**Table 4:** cfDNA concentrations before and after SPRI purification.

Sample	Starting (ng)	cfDNA (ng)	Genomic (ng)	% Recovered (Tot)	% Recovered (cfDNA)	% Recovered (genomic DNA)
C1	44	34	11.8	104.1	74.2	25.8
WT1	50.04	13.6	26.9	80.9	33.6	66.4
WT2	19.8	7.9	7.3	76.8	52.0	48.0
WT3	17.4	7.6	7.4	86.2	50.7	49.3
WT AVG	29.08	9.7	13.9	81.0	45.4	54.6
PDAC1	19.08	8	7.4	80.7	51.9	48.1
PDAC2	27.84	11.3	2.1	48.1	84.3	15.7
PDAC3	74.4	21.8	29.7	69.2	42.3	57.7
PDAC AVG	40.44	13.7	13.1	66.2	59.5	40.5

Based on these results we recommend a careful visual inspection of the peak at ∼10,380 bp because even when it appears that no high molecular weight DNA is present, there could be as much as 66% of contaminating genomic DNA in the cfDNA preparation. The higher percentage of contaminating genomic DNA could be simply a reflection of a longer storage time for the mouse samples between blood draw and processing. This is a recognized problem (Parpart-Li et al., 2016), and the development of collection tubes with additives to prevent contamination of genomic DNA in the plasma are being explored (Norton et al., 2013). Our protocol has the added advantage of a simple bead based purification step to remove any contaminating high molecular weight DNA to ensure that DNA that may result from the lysis of white blood cells during the plasma/serum isolation is eliminated from the preparations. Ultimately, this step greatly increases the sensitivity of our approach for the mapping of tumor-derived DNA methylation differences present in the plasma/serum. In clinical settings, where there is limited control over the lapse in time between blood draw and processing, the genomic DNA contamination may be a significant problem. In addition, the purification step that we propose does not interfere with the blood collection protocols routine in clinical settings.

### Generation of cfDNA Libraries for DNA Methylation Analysis Using NGS Sequencing

**FIGURE 3:**
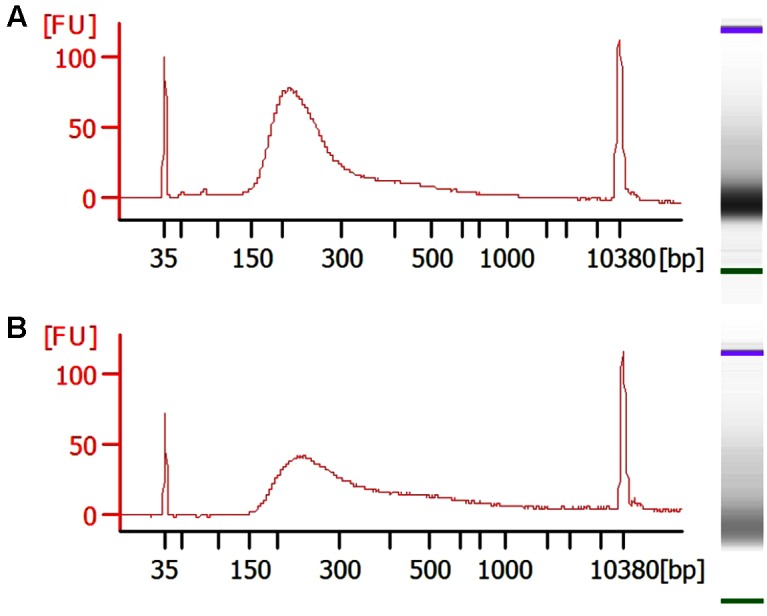
Representative bioanalyzer images of cfDNA libraries. **(A)** Human and **(B)** mouse samples.

Bisulfite converted libraries were generated using the Pico Methyl-Seq Library Prep Kit following the manufacturers instructions. In order to maximize the input DNA, in each reaction we used the maximum volume of DNA that the reaction could accommodate. This volume of 20 μl corresponded to a wide range of total concentrations from 3 to 1,400 ng. In addition, as a positive control we also included one sample of control high molecular weight genomic DNA (100 ng). Libraries were generated from 15 human and 6 mouse samples for a total of 21 samples. After preparation the libraries were analyzed using the bioanalyzer to assess their quality and fragment size. All libraries generated fragment peaks of ∼100–300 bp corresponding to the anticipated size of cfDNA libraries with, as expected, a minor shift of ∼70–150 bp to the right corresponding to the addition of the sequencing adapters. Representative bioanalyzer profiles are shown in **Figure 3**. All libraries passed quality control based on their bioanalyzer profile and quantification of their concentration as assessed by Qubit. All samples were considered suitable for massively parallel sequencing.

### Sequencing Results and Biological Findings

**FIGURE 4:**
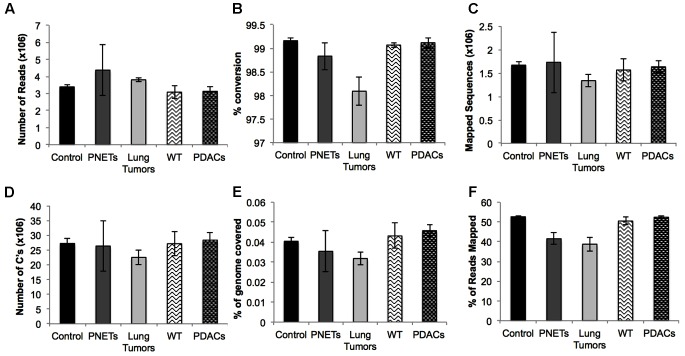
Sequencing results for each sample group. Averages are depicted for: **(A)** the number of reads, **(B)** the percent conversion calculated from percent methylated non-CpGs, **(C)** the number of sequences that uniquely aligned to the reference genome, **(D)** the total number of cytosines evaluated, **(E)** the percent of the total genome with at least 1X coverage, and **(F)** the mapping efficiency of the sequences. Error bars represent standard error of the mean for the individual samples in the group.

**FIGURE 5:**
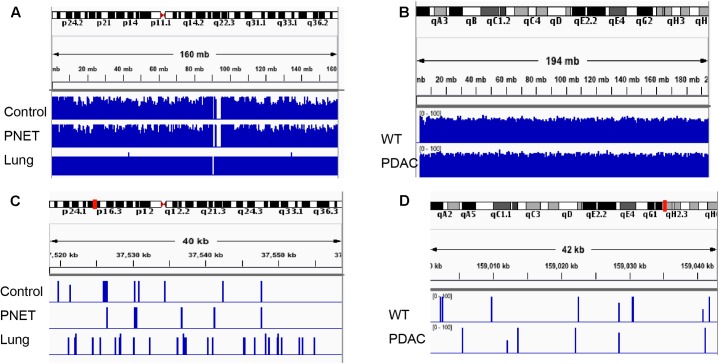
Integrative Genomics Viewer (IGV) visualization depicting representative human and mouse alignments. **(A)** Sequencing reads mapping to human chromosome 2 are visualized at low magnification covering the entire autosome. **(B)** Sequencing reads mapping to the entire mouse chromosome 1. **(C,D)** Depicts a zoomed in area spanning ∼40 kb mapping to the chromosomal region indicated by the red mark on the corresponding autosome ideogram on the top of the panel.

To assess the performance of the cfDNA libraries and to determine if, despite the lower input, the murine libraries performed similar to the one generated with human genomic material we opted to perform the sequencing using the MiSeq sequencer. Because of the relatively low throughput of this instrument we sequenced only five samples from each group. A total of 15 human samples were sequences in three MiSeq lanes and all 6 mouse samples were sequences in one lane. For sequencing we randomly selected control samples 1-2-3-4 and a genomic DNA control, PNET samples 1-2-3-5-7, lung 3-5-6-7-8, and all the mouse samples. The sequencing results were evaluated for further downstream analysis based on read length, number of reads, and quality score across all bases as described in Andrews (2010). All samples passed sequencing quality control with a Phred quality score above 28, read length distributions with peaks at 100 bp, and generated a number of reads that was expected based on the MiSeq throughput and the number of samples that we multiplexed (i.e., greater than 2 × 10^6^ reads per sample). We next proceeded to analyze the sequencing results using the Bismark package (Krueger and Andrews, 2011) that maps bisulfite converted sequences back to the reference genome and provides a methylation call for each cytosine included in the sequence reads. Therefore, number of reads, percent of mapped reads, percent of genome coverage, number of cytosines, conversion rate, and mapping efficiency was calculated for each sample and an average of these variables was determined for each sample group (**Figure 4**). Overall, the sequencing results were highly consistent between sample types and within each group. We obtained approximately 15–25 million reads per lane, an average of 3.8 × 10^6^ reads per sample (minimum: 2.5 × 10^6^, maximum: 11.1 × 10^6^) (**Figure 4A**). The bisulfite conversion efficiency was greater than 97% for all samples analyzed as calculated from the percent methylated non-CpGs (**Figure 4B**). High efficiency of conversion is crucial for a sensitive evaluation of DNA methylation levels. The values that we obtained from cfDNA are in agreement with the conversion rates previously reported using genomic DNA (Holmes et al., 2014). We were able to uniquely map ∼45% of the sequencing reads to their respective reference genomes (average: 1.6 × 10^6^, minimum: 0.71 × 10^6^, maximum: 4.5 × 10^6^) (**Figure 4C**). This mapping efficiency range of 29–54% matches the expected values reported in multiple WGBS studies (Chatterjee et al., 2013; Tran et al., 2014). The number of cytosine evaluated as being either unmethylated or methylated for each sample was ∼25 × 10^6^ (maximum: 62.4 × 10^6^ and minimum: 11.5 × 10^6^). When inspecting the percent of the genome covered we anticipated, based on our multiplexing strategy and the MiSeq throughput (up to 25 million reads per lane when sequencing with the 1 × 100 bp cycles), to be about 0.1%. Indeed, the average coverage of our own sequencing run was about 0.043% (maximum: 0.081%, minimum: 0.018%) (**Figure 4E**) which, based on the calculated mapping efficiency of 45%, indicates that our libraries and sequencing protocol performed as expected. Therefore we conclude that no bias was introduced during the amplification, library preparation or sequencing steps. Based on the analytical output of the sequencing results the libraries generated from the mouse-derived cfDNA were indistinguishable from the human derived samples. Therefore, even when the starting volume of blood is ultralow, the pipeline that we developed produces high quality bisulfite converted libraries for the measurement of DNA methylation changes in mice. These observations were confirmed by visual inspection of the sequencing results using the IGV (Robinson et al., 2011). Sequencing reads uniformly mapped across genomic regions for both human (**Figure 5A**) and murine samples (**Figure 5B**) as visualized for human chromosome 2 and mouse chromosome 1. At higher magnification the sequencing reads were scattered as reflection of low coverage but still uniformly distributed throughout the genome (**Figures 5C,D**). Taken together, our results demonstrate that we were able to generate cfDNA libraries suitable for sequencing for both human and mouse cfDNA after removal of contaminating high molecular weight DNA. In addition we did not observe differences in genome coverage or conversion efficiency due to the low input even when starting with as little as 3 ng of cfDNA as input.

### DNA Methylation Differences Can Be Identified in Both Human and Mouse cfDNA Derived from Tumor Samples Relative to Non-tumor Controls

This project was designed to develop a protocol for the efficient preparation and sequencing of bisulfite converted cfDNA libraries for high throughput analysis. Because we opted to analyze our samples using the MiSeq sequencer the throughput was low, generating ultra low coverage (∼0.05X). Therefore, we are unable to identify with high confidence biologically significant DMRs between tumor derived cfDNA and control samples based on these sequencing results. Yet, even with ultra low coverage we were able to pinpoint to regions of differential methylation (DMRs) suggesting that cfDNA methylation differences exist, for humans and murine models of human disease.

**FIGURE 6:**
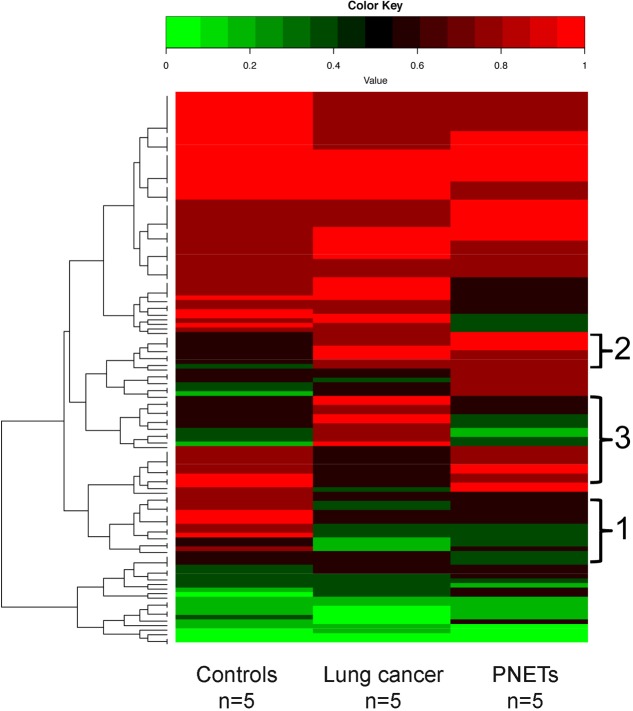
DNA methylation profile of human cfDNA. The averages of the DNA methylation levels within each group detected in all CpG sites that were common amongst the human samples is plotted for controls, lung cancer patients and PNET patients. The rows indicate individual CpG sites with each row showing the average methylation value of that site in each sample type. Red represents high methylation levels and green low methylation levels. Color scale with methylation levels is depicted above the heatmap. Region 1 in brackets indicates cfDNA hypermethylation in controls relative to tumors; region 2 indicates hypermethylated cfDNA regions in tumors relative to controls; region 3 indicates cfDNA methylation differences between the lung and PNETs samples.

A heat map depicting the cfDNA methylation levels measured across all sequenced CpG sites that were in common between all human samples was generated (**Figure 6**). As expected, most of the regions are methylated (Bird, 2002; Board et al., 2008). The average of methylation levels between the three human groups (controls, lung cancer patients, and PNETs) clearly reveals that cfDNA methylation differences exist (**Figure 6**).

In the human dataset, we identified 133 sequenced CpGs that mapped to chromosome positions covered in all 15 samples analyzed (Supplementary Table S1). Due to low coverage only chromosomes 1, 3, 5, 7, 14, 16, 17, 18, 19, 21, and 22 had sequencing regions that were found present in all samples. When analyzed for genomic compartments only two sequenced CpGs mapped to gene bodies associated with UNIPROT identifiers [Mitochondrially Encoded NADH:Ubiquinone Oxidoreductase Core Subunit 5 (MT-ND5) and Mitochondrially Encoded Cytochrome B (MT-Cyb)]. Seven sequencing fragments mapped to shore regions; all other CpGs mapped to gene deserts. One most likely explanation for the low correlation between sequenced regions and other functional genomic compartments is low coverage. However, WGBS of non-small lung cancer previously reported a significant enrichment of hypo-methylated regions that did not correlate to any particular functional category of genes (Carvalho et al., 2012; Mullapudi et al., 2015).

Of the commonly identified CpGs 42 were hyper-methylated and 45 were hypo-methylated in the lung cancer samples (at least 20% differential methylation) relative to controls; while 36 CpGs were hyper-methylated and 54 were hypo-methylated in the PNET samples. Of these DMRs, 43 were hypo-methylated in both cancer datasets (**Figure 6**, region 1), and 20 were hyper-methylated in both lung and PNETs relative to controls (**Figure 6**, region 2). When analyzed in terms of tumor type specific DMRs we found that 17 CpGs were uniquely hyper-methylated and 4 were hypo-methylated in the lung cancer patients (at 20% differential methylation levels). In terms of PNETs specific DMRs, we found that 8 CpGs were hyper-methylated and 6 were hypo-methylated in this cancer type (**Figure 6**, region 3). The tumor specific differences suggest that biological differences between tumor subtypes at the DNA methylation level are reflected in cfDNA.

**FIGURE 7:**
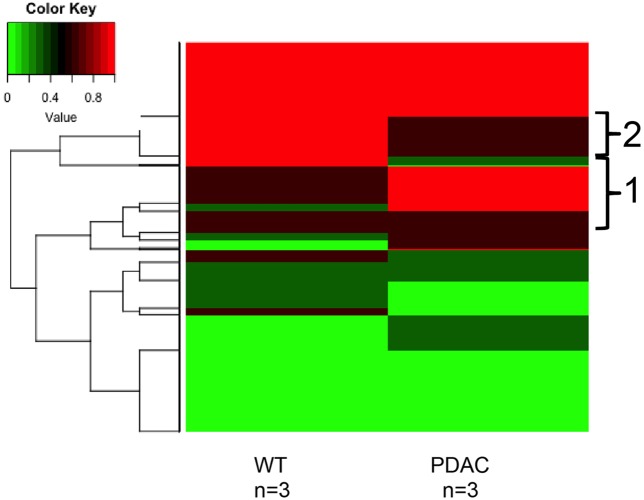
DNA methylation profile of murine cfDNA. The averages of the DNA methylation levels detected in all CpG sites common amongst all the murine sample analyzed is plotted for WT animals and the PDAC murine model. Red represents high methylation levels and green low methylation levels. Color scale with methylation levels is depicted above the heatmap. Region 1 in brackets indicates hypermethylation in PDAC cfDNA; region 2 indicates hypermethylation in the WT cfDNA relative to PDAC samples.

Likewise, a similar analysis to detect cfDNA methylation differences was performed using the sequencing results obtained from the murine WT vs. PDACs samples (**Figure 7**). We found 1150 sequenced CpGs that mapped to chromosome positions covered in all 6 samples analyzed; we therefore proceeded to investigate these sequences in more detail (Supplementary Table S1). All mouse chromosomes contained sequenced CpGs common to all samples, yet Chr17 was the most enriched (413 CpGs). This is particularly interesting since mouse Chr17 is a gene rich chromosome and it is especially enriched for oncogenes and tumor suppressor genes (Montagna et al., 2002, 2003; Weaver et al., 2002; Dorritie et al., 2004; Ried et al., 2004; Wang et al., 2007). In the PDAC samples 290 CpGs were hyper-methylated and 281 were hypo-methylated. Of the common sequenced CpGs 88 mapped to gene bodies of which 66 (mapping to 16 genes) where differentially methylated between PDACs and controls. Gene ontology analysis of the DMRs mapping to the gene bodies identified a significant enrichment (*p* < 1e-3) within the Biological General Repository for Interaction Datasets (BioGRID) of autophagy (Autophagy Related 7, *Atg7*). Of note, an essential role for autophagy in PDAC growth and survival has recently emerged (New et al., 2017), and the autophagy essential gene *Atg7* has been shown to fuel tumor growth in mice containing oncogenic Kras (the most common mutation event in human PDACs) (Rosenfeldt et al., 2013). While the analysis of additional biological replicate samples sequenced at higher coverage is required to draw robust conclusions, the heat map visualization indicates that cfDNA methylation differences can be identified also in the murine cfDNA using the approach here described. In fact, region 1 in **Figure 7** pinpoints to cfDNA that is hypermethylated in PDACs samples while region 2 pinpoints to genomic regions of cfDNA hypomethylation.

## Discussion

We have described a new method for cfDNA isolation and library generation for use in genome wide DNA methylation profiling of both murine and human samples. Though WGBS has been performed using human cfDNA as the input in the past, to our knowledge, this is the first time that this protocol has been performed on murine samples. That DNA methylation profiling of cfDNA holds the potential of a sensitive biomarker is suggested by WGBS of cfDNA of pregnant women where placenta specific DNA methylation regions could be detected in the blood of the mothers (Jensen et al., 2015). Chan et al. (2013) have demonstrated that low coverage WGBS of cfDNA is sufficient to detect overall hypo- versus hyper-methylated cfDNA in a variety of cancer patients. In addition, for hepatocellular carcinomas patients, even though the sample size was small (*n* = 2), the authors detected no substantial global cfDNA methylation changes post-surgery in a metastatic patient while significant cfDNA loss of hypo-methylation was found in the disease free survivor. Other studies have investigated the potential of DNA methylation analysis of cfDNA to stratify high versus low risk cancer patients using targeted DNA methylation profiling of small gene sets (Legendre et al., 2015), or PCR based methods for the sensitive detection of DNA methylation changes in cfDNA at predetermined loci in lung cancer patients (Liu et al., 2016). However none of the previously reported studies investigated the potential contribution of DNA methylation changes in cfDNA from high molecular weight DNA, and whole genome profiling at the single locus analysis remains unexplored. Likewise, to our knowledge, the use of murine models to profile cfDNA methylation changes has remained unexplored.

We were able to successfully generate and sequence libraries from cfDNA from both human and mouse blood samples. The samples showed consistent high quality control parameters as assessed by the percent of conversion and the percent of mapped reads across all samples. The individual sample-to-sample variability of sequencing parameters was fairly minimal and the differences were not statistically significant. There was however, substantial variability in the starting concentrations of cfDNA. Due to the low number of samples analyzed and the high variability in the patients with tumors, there were no statistically significant cfDNA concentration differences between the tumor cohorts and the control group. However, a statistical significant higher cfDNA blood concentration was observed in PNETs compared to lung cancer patients. PNETs cfDNA was serum derived, and because the yield of cfDNA isolated from serum has been reported higher than plasma (Lee et al., 2001) it is possible that these differences are due to the source of starting material rather than intrinsic biological differences between tumor types. Other general trend differences were apparent. In the mouse samples, the PDAC mice had slightly higher but not significant cfDNA concentration than the WT. The increased yield of cfDNA in murine models compared to human samples was previously reported (Cheng et al., 2009; Cortese et al., 2015; Hamaguchi et al., 2015), however, a biological explanation for the roots of the differences in cfDNA biology between the two mammalian species remains unknown. Based on the observed variability between samples in the yield of cfDNA, the analysis of a larger number of samples is necessary to draw conclusions.

Sequencing at high depth is expected to generate valuable and meaningful biologically relevant results. When our data was compared to that of Legendre et al. (2015) who, like us, used Bismark for alignment but a different library generation kit (Methyl-Seq Library System from NuGen) and the same cfDNA isolation protocol, without the AMPure bead purification, we obtained similar percentages of mapped, usable reads (45–55% of starting reads vs. 29–53%, respectively). Producing bisulfite converted libraries suitable for NGS analysis is challenging when cfDNA is used as input, because of the low starting material and the fragmented DNA. While we developed the protocol presented here for WGBS, the additional purification step can be adopted for other cfDNA sequencing approaches (i.e., targeted sequencing) and may help increase sensitivity.

We have also demonstrated that the method described here is suitable for the analysis of ultra low DNA input (∼3 ng) and can be applied for the cfDNA methylation profiling of murine models for human disease providing unprecedented tools to study blood based biomarkers to monitor disease progression and therapy response. Application of our proposed experimental pipeline to murine models of cancer has also the potential to address biological questions beyond the more straightforward biomarker discovery studies. Fundamental unanswered biological questions about mechanisms of cfDNA sheading into the blood stream, tumor subtype variation of cfDNA released, how tumor heterogeneity is reflected at the cfDNA level and how it varies in respond to therapy can most benefit from murine studies.

## Ethics Statement

This study was carried out in accordance with the recommendations of the Albert Einstein College of Medicine Committee on Clinical Investigations with written informed consent from all subjects. All subjects gave written informed consent in accordance with the Declaration of Helsinki. The protocol was approved by the Albert Einstein College of Medicine Committee on Clinical Investigations (CCI#2007–433 and 09-06-173). This study was carried out in accordance with the recommendations of the Institutional Animal Care and Use Committee (IACUC). The protocol was approved by the Albert Einstein IACUC.

## Author Contributions

EM and SG carried out the experimental work including data analysis and drafted the manuscript. HC and BP enrolled the patients and provided the samples for this study. ZY acquired the mouse samples for this study. XD developed the analytical pipeline for data analysis and assisted in analysis. SL and JV contributed to the study design. CM designed and supervised the study. HC, BP, ZY, SL, JV, and CM critically edited the manuscript.

## Conflict of Interest Statement

The authors declare that the research was conducted in the absence of any commercial or financial relationships that could be construed as a potential conflict of interest.

## Footnotes

1 https://www.ncbi.nlm.nih.gov/sra
