# Supplementary material for: Development of a Method to Implement Whole-Genome Bisulfite Sequencing of cfDNA from Cancer Patients and a Mouse Tumor Model
Source: Front Genet. 2018 Jan 23;9:6. doi: 10.3389/fgene.2018.00006 (PMC5787102; doi:10.3389/fgene.2018.00006)
Supplement: Supplementary file 2 [file Table_2.DOCX]

**Detailed Protocol**

1. Collect blood in EDTA tubes and spin at 2000 x g in a 4°C centrifuge to separate plasma.
   1. Alternative for serum: collect blood in Clot activator and gel for serum separation (red top venous puncture tubes).
   2. Incubate at room temperature for ~30 min to allow blood clotting
2. Immediately remove plasma or serum layer from the tube and proceed with cfDNA isolation or freeze at -80°C.

**Isolation of cfDNA**

1. Accurately measure starting plasma volume and continue with kit instructions of the Qiagen QIAamp Circulating Nucleic Acid kit (Cat No./ID: 55114) up to step 10.
2. Add an additional wash with Wash Buffer 1 of 200 μl then continue with protocol.
3. Heat elution buffer to 40 °C before use.
4. Elute in two elution steps (50 μl followed by 30 μl) with pre-warmed elution buffer to ensure maximum recovery.
5. Combine the two eluted fractions.

**Removal of High Molecular Weight DNA**

1. Remove AMpure beads from the 4°C refrigerator and allow to warm to room temperature (RT) before use.
2. Add 0.5x volume of beads to the cfDNA.
3. Mix well by pipetting up and down.
4. Incubate at RT for 5 min.
5. Place tubes on magnetic rack to bind the beads. Once bound transfer the supernatant to a new sterile eppendorf tube.
6. Add 1.6x volume of beads.
7. Mix well by pipetting up and down.
8. Incubate at RT for 5-10 min.
9. Place the tube on a magnetic rack to bind the beads then remove and discard the supernatant.
10. Immediately add 200 μl of freshly prepared 80% ethanol while the tube is in the magnet.
11. Incubate at RT for 30 sec.
12. Remove ethanol.
13. Repeat the 80% ethanol wash step.
14. Allow the pellet to dry on the magnet just until it has a slightly matte appearance. Do not over-dry the beads!
15. Remove the eppendorf tube from the magnet.
16. Resuspend in elution buffer (10 mM Tris-HCl, 0.1 mM EDTA, pH 8.5).
17. Incubate at RT fro 2 min.
18. Place the eppendorf tube in magnet until solution clears.
19. Transfer to a new eppendorf tube being careful not to transfer any beads.

NOTE: micro-capillary based electrophoretic analysis (like the Bioanalyzer) is recommended at all stages of high molecular weight DNA removal to carefully analyze the size distribution of DNA fragments.

**Library Generation**

1. Quantify cfDNA concentration using the Qubit™ dsDNA HS Assay.
2. Follow instructions as directed in the Pico Methyl-Seq™ Library Prep Kit. Notes: Use 15 min incubation in Section 1, Step 5. When adjusting cycle number in Section 4 add an additional 2 cycles to recommended amount (up to 10 cycles).
